# Sugammadex, neostigmine and postoperative pulmonary complications: an international randomised feasibility and pilot trial

**DOI:** 10.1186/s40814-021-00942-9

**Published:** 2021-11-09

**Authors:** Kate Leslie, Matthew T. V. Chan, Jai N. Darvall, Anurika P. De Silva, Sabine Braat, Nancy J. Devlin, Philip J. Peyton, Jade Radnor, Carmen K. M. Lam, Sofia Sidiropoulos, David A. Story

**Affiliations:** 1 Department of Critical Care, Melbourne Medical School, University of Melbourne, Melbourne, Australia; 2 Department of Anaesthesia and Pain Management, Royal Melbourne Hospital, Melbourne, Australia; 3 Department of Anaesthesia and Intensive Care, Chinese University of Hong Kong, The Prince of Wales Hospital, Hong Kong Special Administrative Region, People’s Republic of China; 4 Centre for Epidemiology and Biostatistics, Melbourne School of Population and Global Health, University of Melbourne, Melbourne, Australia; 5 Methods and Implementation Support for Clinical and Health (MISCH) Research Hub, Faculty of Medicine, Dentistry and Health Sciences, University of Melbourne, Melbourne, Australia; 6 Health Economics Unit, Melbourne School of Population and Global Health, Melbourne, Australia; 7 Department of Anaesthesia, Austin Health, Melbourne, Australia; 8 Department of Anaesthesia and Pain Management, Northeast Health Wangaratta, Wangaratta, Australia; 9 Department of Anaesthesia and Operating Room Services, Tuen Mun Hospital, Hong Kong Special Administrative Region, People’s Republic of China

**Keywords:** Sugammadex, Neostigmine, Neuromuscular blockade, Atelectasis, Pneumonia

## Abstract

**Background:**

Sugammadex reduces residual neuromuscular blockade after anaesthesia, potentially preventing postoperative pulmonary complications. However, definitive evidence is lacking. We therefore conducted a feasibility and pilot trial for a large randomised controlled trial of sugammadex, neostigmine, and postoperative pulmonary complications.

**Methods:**

Patients aged ≥40 years having elective or expedited abdominal or intrathoracic surgery were recruited in Australia and Hong Kong. Perioperative care was at the discretion of clinicians, except for the use of rocuronium and/or vecuronium for neuromuscular blockade and the randomised intervention (sugammadex or neostigmine) for reversal. Feasibility measurements included recruitment, crossover, acceptability, completeness, and workload. Trial coordinator feedback was systematically sought. Patient-reported quality of life was measured using the EQ-5D-5L score. The primary pilot outcome was the incidence of new pulmonary complications up to hospital discharge (or postoperative day 7 if still in hospital).

**Results:**

Among 150 eligible patients, 120 consented to participate (recruitment rate 80%, 95% confidence interval [CI] 73 to 86%). The randomised intervention was administered without crossover to 115 of 117 patients who received reversal (98%, 95% CI 94 to 100%). The protocol was acceptable or highly acceptable to the anaesthetist in 108 of 116 cases (93%, 95% CI 87 to 97%; missing = 4). Four patients of the 120 patients were lost to follow-up at 3 months (3.3%, 95% CI 0.9 to 8.3%). Case report forms were complete at 3 months for all remaining patients. The median time to complete trial processes was 3.5 h (range 2.5–4.5 h). Trial coordinators reported no barriers to trial processes. Patients were aged 64 (standard deviation 11) years, 70 (58%) were male and 50 (42%) were female, and planned surgeries were thoracic (23 [19%]), upper abdominal (41 [34%]), and lower abdominal (56 [47%]). The primary outcome was observed in 5 (8.5%) of the 59 sugammadex patients and 5 (8.2%) of the 61 neostigmine patients (odds ratio 1.02, 95% CI 0.28 to 3.67).

**Conclusions:**

A large international randomised controlled trial of sugammadex, neostigmine and postoperative pulmonary complications in adult patients having abdominal and intrathoracic surgery, including collection of cost-effectiveness evidence for Health Technology Appraisal, is feasible.

**Trial registration:**

Prospectively registered at the Australian and New Zealand Clinical Trials Registry (ACTRN12620001313921) on December 7, 2020. www.anzctr.org.au/Trial/Registration/TrialReview.aspx?id=380645&isReview=true.

**Supplementary Information:**

The online version contains supplementary material available at 10.1186/s40814-021-00942-9.

## Key messages

1. What uncertainties existed regarding the feasibility?
  The acceptability of randomisation to sugammadex or neostigmine for patients and their anaesthetists was unclear. We were also uncertain about the acceptability of randomisation at the end of surgery and whether anaesthetists would administer the randomised intervention without crossover. The barriers to site initiation, screening, recruitment and data collection, including patient-reported quality of life, were unknown.
2. What are the key feasibility findings?
  The recruitment rate (80%) and rate of administration of the randomised intervention (98%) without crossover were high. Four of the 120 patients were lost to follow-up at 3 months. Case report forms were complete at 3 months in the remaining patients. The protocol was acceptable to the anaesthetist in 93% of cases. The median time taken for trial procedures was 3.5 h. No specific barriers to initiation, screening, recruitment or data collection were encountered.
3. What are the implications of the feasibility findings for the design of the main study?
  We demonstrated the feasibility of a large international randomised trial of sugammadex, neostigmine and postoperative pulmonary complications in adult patients having abdominal and intrathoracic surgery, including collection of cost-effectiveness evidence for Health Technology Appraisal. Opportunities to improve trial processes, including improved anaesthetist and trial coordinator initiation, were identified.

## Background

Sugammadex is a novel cyclodextrin agent for reversal of neuromuscular blockade produced by aminosteroid neuromuscular blocking drugs [1]. Sugammadex rapidly and effectively reverses even deep neuromuscular blockade, preventing residual neuromuscular blockade as patients emerge from general anaesthesia and potentially reducing the risk of airway obstruction, aspiration of gastric contents, pulmonary atelectasis and pneumonia [2]. These complications often lead to prolonged hospital stay, increased mortality and decreased patient-reported quality of life [3, 4], but few preventive strategies are available [5]. Unwanted effects of sugammadex include interactions with hormonal contraceptives [6] and anaphylaxis [4], and it remains expensive relative to standard anticholinesterase reversal agents [7].

Interest in proving whether sugammadex can prevent postoperative pulmonary complications is therefore strong [8]. Numerous observational studies and a handful of small randomised trials [9–12] compared postoperative pulmonary complications after post-anaesthesia care unit discharge in adult patients receiving sugammadex or an anticholinesterase. In the largest observational study (*n*=45,712), sugammadex was associated with fewer postoperative pulmonary complications than neostigmine at hospital discharge (adjusted odds ratio [aOR] 0.70, 95% confidence interval [CI] 0.63 to 0.77) [13], whereas the second largest (*n*=8,795) found no difference (aOR 1.03, 95% CI 0.85 to 1.25) [14]. The four randomised controlled trials [9–12] included 50 events in 284 sugammadex patients (18%) and 68 events in 278 neostigmine patients (24%) (relative risk 0.77, 95% CI 0.57 to 1.04, random effects model). A survey of anaesthesia clinical trialists revealed that 89% consider postoperative pulmonary complications to be important and 62% agree that a large randomised controlled trial comparing sugammadex with neostigmine is worthwhile [15].

We therefore conducted a randomised feasibility and pilot trial aiming to determine the feasibility of a large international randomised controlled trial of sugammadex, neostigmine and postoperative pulmonary complications in adult patients having abdominal and intrathoracic surgery.

## Methods

### Trial design

We conducted a multi-centre randomised patient- and observer-blinded feasibility and pilot trial at three sites in Victoria, Australia, and two sites in Hong Kong, People’s Republic of China. Ethics approval was obtained at each site, and the trial was registered at the Australian and New Zealand Clinical Trials Registry (ACTRN12620001313921) before recruitment began. Patients were enrolled preoperatively after providing written informed consent. The trial conforms with the Consolidated Standards of Reporting Trials guidelines (see Additional Information). There were no changes to the protocol after the trial began.

### Trial aims

Our aims were to determine the following in patients aged 40 years and over having abdominal and thoracic surgery under general anaesthesia who were randomised to sugammadex or neostigmine for reversing neuromuscular blockade.

### Feasibility aims

1. Rate of recruitment of eligible patients who were approached for consent to participate
2. Proportion of administration of the randomised intervention without crossover to the alternative intervention
3. Acceptability of the protocol to the anaesthetist
4. Completeness of patient follow-up at 3 months
5. Completeness of the case report form at 3 months
6. Time taken to complete all trial procedures
7. Views of trial coordinators on recruitment, data collection and follow-up.

### Pilot aims

1. Incidence of new pulmonary complications up to hospital discharge (or postoperative day 7 if still in hospital) using the Standardised Endpoints for Perioperative Medicine - Core Outcome Measures in Perioperative and Anaesthetic Care definition [16] (see Additional Information).
2. Incidence of atelectasis until hospital discharge (or postoperative day 7 if still in hospital)
3. Incidence of pneumonia until hospital discharge (or postoperative day 7 if still in hospital)
4. Incidence of acute respiratory distress syndrome until hospital discharge (or postoperative day 7 if still in hospital)
5. Incidence of pulmonary aspiration until hospital discharge (or postoperative day 7 if still in hospital)
6. Incidence of postoperative nausea and vomiting on postoperative day 1
7. Incidence of unplanned intensive care unit/high dependency unit admission until hospital discharge
8. Days alive and at home on postoperative day 30
9. Health-related quality of life at 3 months postoperatively, as measured by EQ-5D-5L [17]
10. Duration of post anaesthesia care unit stay
11. Incidence of airway instrumentation in post anaesthesia care unit
12. Quality of recovery on postoperative day 1, as measured by the Quality of Recovery 15-item (QoR-15) score [18]
13. Frailty at 3 months postoperatively, defined by a Clinical Frailty Scale (CFS) score >4 [19].

### Patient population

Patients were eligible if they were aged 40 years and older and having elective or expedited intraabdominal, retroperitoneal, pelvic and non-cardiac intrathoracic surgery under relaxant general anaesthesia with an endotracheal tube, expected to last ≥2 h and with an expected hospital stay of ≥1 postoperative night. Patients were excluded if they were unable to provide written informed consent (e.g., language barrier, intellectual disability, cognitive deficit, urgent surgery) or if there was a plan for a skin incision and/or vascular access at or below the inguinal ligament without an abdominal or thoracic skin incision, intraoperative administration of neuromuscular blocking drug other than rocuronium or vecuronium, reversal of neuromuscular blockade during surgery, spontaneous complete recovery from neuromuscular blockade during surgery, a contraindication to sugammadex or neostigmine, elective postoperative invasive ventilation or previous randomisation to the trial.

Patients were screened for eligibility preoperatively by trial coordinators, using medical records and surgical booking lists. Sites maintained screening logs documenting reasons for not consenting and randomising eligible patients.

### Randomisation and blinding

Patients were randomly assigned on a 1:1 basis to sugammadex or neostigmine. The randomisation list was stratified by region and within region, by site. The randomisation list was computer-generated by an independent statistician using randomly permuted blocks. Randomisation results were concealed until the end of surgery. All randomised patients remained in the group to which they were assigned, on an intention-to-treat basis, and were followed until the end of the trial unless they withdrew consent. Patients, observers, endpoint adjudicators, the trial statistician and the trial health economist were blind to group assignment. The anaesthetists were blind to group assignment until they were advised of the randomisation by an unblinded investigator or trial coordinator at the end of surgery. The aim of this timing was to avoid biased anaesthetic management and to incentivise careful management of neuromuscular blockade.

### Trial interventions

Preparations of sugammadex and neostigmine that were available at participating sites were prepared and administered by the anaesthetists as a single intravenous dose at the end of surgery. Dosage was at the anaesthetist’s discretion (see below). Neostigmine was administered with the anaesthetist’s choice of intravenous atropine or glycopyrrolate.

### Patient care

All preoperative and postoperative care was at the discretion of the treating team. Intraoperative care was at the discretion of the treating team, except for choice of neuromuscular blocking drug and reversal drug. Neuromuscular blockade was achieved with rocuronium and/or vecuronium only, with succinylcholine at induction, if indicated. Neuromuscular blockade was reversed at the end of surgery with the randomised intervention, and the endotracheal tube was removed before the patient left the operating room. Further (‘rescue’) reversal was administered at the discretion of the anaesthetist. Timing, dosage and monitoring of neuromuscular blocking drugs, the randomised intervention and rescue reversal drugs were at the discretion of the anaesthetist. Anaesthetists were advised to refer to the product information, expert guidelines and the results of quantitative neuromuscular monitoring to guide maintenance and reversal of neuromuscular blockade. If a decision was made during surgery to reverse neuromuscular blockade and extubate the trachea after the patient left the operating room, the randomised intervention was not administered, and further management was at the discretion of the treating team.

### Data collection

Data were collected from patients and their medical records by blinded trial coordinators. Assess Respiratory Risk in Surgical Patients in Catalonia (ARISCAT) scores were calculated from preoperative characteristics and were classified into low (<26), intermediate (26–44) or high risk (>44) [4]. Apfel scores were calculated to assess risk of postoperative nausea and vomiting (high risk = 3–4) [20, 21]. Safety outcomes, including infectious, cardiovascular, thromboembolic, respiratory, neurological, digestive, renal, musculoskeletal and allergic untoward events, were collected from randomisation until hospital discharge (or postoperative day 7 if still in hospital). Other untoward events were defined as adverse events and were collected from randomisation until 3 months postoperatively.

Anaesthetists were interviewed about the acceptability of the protocol in the post anaesthesia care unit. Thirty-day and 3-month patient follow-ups were conducted by telephone unless the patient was at the trial site for clinical care. The trial coordinators were interviewed about their experiences at the end of the trial using a standardised questionnaire. Study data were collected and managed using REDCap electronic data capture tools hosted by the University of Melbourne. There were no changes to data collection during the trial.

### Sample size

Assuming 60 patients were recruited to each group, if a successful intervention delivery rate of 90% was observed, then the two-sided 95% CI of the true underlying successful delivery rate would be 79 to 96% using the exact (Clopper-Pearson binomial) method. With 120 patients in total, a 3-month follow-up rate of 90% would have a 95% CI of 83 to 95%. The probability of observing at least one safety outcome with 60 patients per study arm is 95% if the underlying event rate is 5% and 100% if the underlying event rate is 10%.

### Statistical analyses

A statistical analysis plan was finalised before unblinding of the database. The analysis set included all randomised patients for the feasibility and pilot outcomes, except for the feasibility outcome on recruitment rate, which included all patients with all inclusions and no exclusions who were approached for consent.

### Feasibility data

Acceptability was dichotomised (‘low’ = highly unacceptable, unacceptable, neutral; ‘high’ = acceptable, highly acceptable). Recruitment rate, rate of administering the randomised intervention without crossover, acceptability rate, 3-month follow-up rate and complete case report form rate are presented as number and percentage of patients with two-sided 95% CIs calculated using the exact (Clopper-Pearson binomial) method. Except for the rate of administering the randomised intervention without crossover, which is presented by randomised group, results of the feasibility outcomes are presented for all randomised patients combined.

### Pilot data

Baseline, surgical, anaesthetic, post anaesthesia care unit and postoperative data were summarised by the randomised intervention using mean (standard deviation) and median (interquartile range) for continuous data, and frequency (percentage) for categorical data. The estimate of the treatment effect (OR or absolute mean or median difference as appropriate), 95% CI and *p* value were obtained using pre-specified methods and were adjusted for site. Firth logistic regression was employed for the primary outcome, and for frailty at 3 months (also adjusted for preoperative status), given the low number of events [22]. Logistic regression was employed for postoperative nausea and vomiting on day 1. Bootstrapped quantile regression with 100 replications was employed for duration of post anaesthesia care unit stay and days alive and at home on postoperative day 30. Linear regression was employed for quality of recovery on postoperative day 1 (also adjusted for preoperative status). The remaining secondary outcomes were not compared between groups using statistical testing. Safety outcomes and adverse events were summarised by treatment group. No adjustment for multiple testing was conducted due to the exploratory nature of the pilot outcomes. In addition to *p* values, effects were interpreted based on the magnitude and direction of the effect along with two-sided 95% CIs (where applicable). No subgroup analyses were conducted due to the small sample size. All descriptive statistics and statistical analyses were based on available data. All statistical analyses were conducted using Stata 16.1 (Stata Corporation, College Station, TX, USA).

Patients’ self-reported EQ-5D-5L data were summarised using relevant value sets and their self-assessed overall health on the EuroQol-Visual Analogue Scale (EQ-VAS) scores were summarised by the randomised intervention using median (interquartile range). EQ-5D-5L index values for Hong Kong were computed using the Hong Kong value set [23]. EQ-5D-5L index values for Australia were computed using the USA value set, in the absence of an Australian value set [24]. As a sensitivity analysis, the EQ-5D-5L index values for Australia were also computed using the English value set [25]. No further analyses were conducted due to the small sample size and primary aim of this study, which was to investigate feasibility.

## Results

**Fig. 1:**
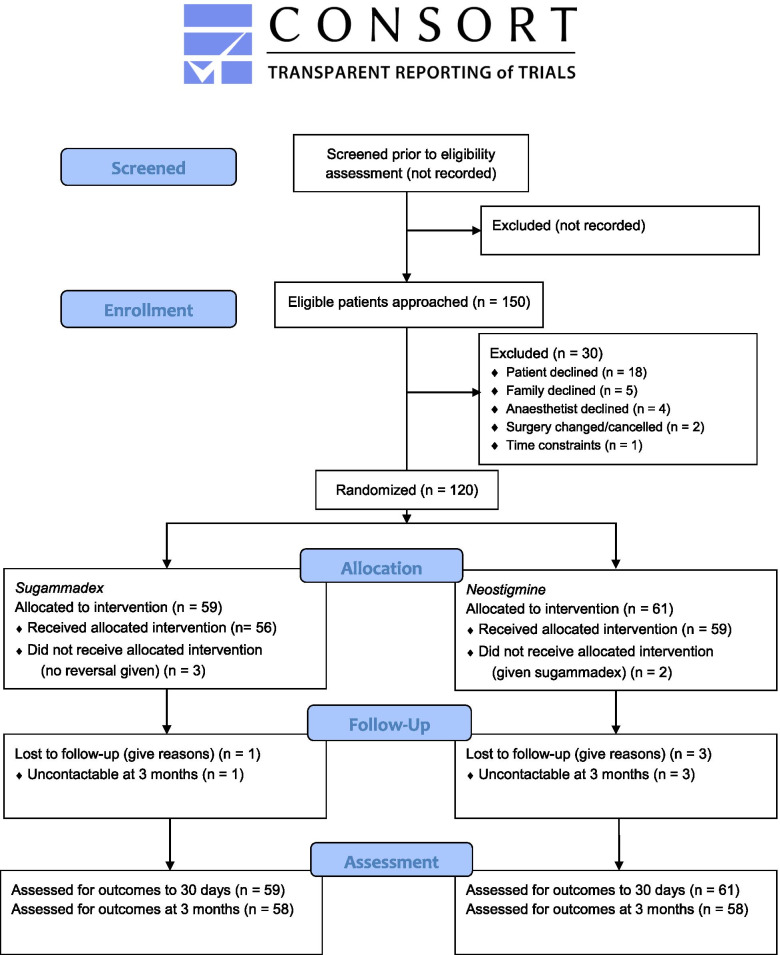
SNaPP pilot consort flowchart

120 patients were recruited between January 15, 2021, and May 20, 2021 (7 patients per week), with a 3-month follow-up completed on August 20, 2021. The flow diagram is presented in Fig. 1.

### Feasibility outcomes

Among 150 eligible patients, 120 consented to participate (recruitment rate 80%, 95% CI 73 to 86%). Reasons for failure to consent eligible patients are included in Fig. 1. The randomised intervention was administered without crossover to 115 of the 117 patients who received reversal (98%, 95% CI 94 to 100%). The protocol was acceptable or highly acceptable to the anaesthetist in 108 of 116 cases where this was measured (93%, 95% CI 87 to 97%). Four patients were lost to follow-up at 3 months (3.3%, 95% CI 0.9 to 8.3%). Case report forms were complete at 3 months in the remaining cases. The median time taken to screen and recruit patients, perform trial procedures and complete all data entry was 3.5 h (range 2.5–4.5 h). The views of trial coordinators about recruitment, data collection and follow-up are presented in the Additional Information. Trial processes presented no specific barriers. Some anaesthetists wanted to know the assigned group at the beginning of the surgery instead at wound closure. The trial coordinators also suggested more training about frailty assessment and neuromuscular monitoring.

### Characteristics of randomised patients

Baseline characteristics are presented in Table 1 and the Additional Information. Patients were aged 64 (11) years, 70 (58%) were male and 50 (42%) were female, and 46 (38%) were classified as American Society of Anesthesiologists physical status 3 or 4. ARISCAT scores indicated an intermediate or high risk of postoperative pulmonary complications in 84 (70%) patients. Apfel scores indicated a high risk of postoperative nausea and vomiting in 52 (43%) patients.

**Table 1:** Baseline characteristics

Characteristic	Sugammadex (***n*** = 59)	Neostigmine (***n*** = 61)
Age (years)	64.6 (11.1)	62.7 (10.1)
Sex		
Male	33 (55.9)	37 (60.7)
Female	26 (44.1)	24 (39.3)
Body mass index (kg/m^2^)	25.6 (4.6)	27.0 (5.8)
ASA physical status		
1	1 (1.7)	5 (8.2)
2	35 (59.3)	33 (54.1)
3	22 (37.3)	22 (36.1)
4	1 (1.7)	1 (1.6)
History of PONV or motion sickness	13 (22.0)	5 (8.2)
Apfel simplified risk score		
1	3 (5.1)	6 (9.8)
2	28 (47.5)	31 (50.8)
3	18 (30.5)	21 (34.4)
4	10 (16.9)	3 (4.9)
ARISCAT score	38 (26–42)	34 (26–41)
ARISCAT risk		
Low (<26)	16 (27.1)	20 (32.8)
Intermediate (26–44)	29 (49.2)	34 (55.7)
High (>44)	14 (23.7)	7 (11.5)
QoR-15 score	136.0 (123.0–144.0)	141.0 (132.0–144.0)
EQ-5D-5L score	0.9 (0.8–1.0)	0.9 (0.9–1.0)
EQ-VAS score	80.0 (75.0–90.0)	80.0 (75.0–90.0)
Frailty		
Non-frail (CFS score 1–4)	57 (96.6)	59 (96.7)
Frail (CFS score 5–9)	2 (3.4)	2 (3.3)

Results are presented as mean (standard deviation), median (interquartile range), and number (percent). *ARISCAT* Assess Respiratory Risk in Surgical Patients in Catalonia, *ASA* American Society of Anesthesiologists, *CFS* Clinical Frailty Scale, *EQ-5D-5L* EuroQol 5-Dimension 5-Level, *EQ-VAS* EuroQol–Visual Analogue, *PONV* postoperative nausea and vomiting, *QoR* quality of recovery, missing data: baseline EQ-VAS = 1 (0.8%)

Intraoperative characteristics are presented in Table 2 and the Additional Information. Thoracic, upper abdominal and lower abdominal surgeries were planned in 23 (19%), 41 (34%) and 56 (47%) patients, respectively. Prophylactic antiemetics were administered to 104 (87%) patients. Quantitative neuromuscular monitoring was applied in 54 (45%) of patients. Of patients allocated to sugammadex (*n* = 59), two were not extubated in the operating room and one spontaneously recovered to a train of four ratio of >0.9. Of patients allocated to neostigmine (*n* = 61), two received sugammadex instead. Further reversal was administered to 9 (7.7%) patients (all in the neostigmine group).

**Table 2:** Intraoperative characteristics

Characteristic	Sugammadex (***n*** = 59)	Neostigmine (***n*** = 61)
Surgical urgency		
Elective	58 (98.3)	60 (98.4)
Expedited	1 (1.7)	1 (1.6)
Surgical incision		
Thoracic	13 (22.0)	10 (16.4)
Upper abdominal	19 (32.2)	22 (36.1)
Lower abdominal	27 (45.8)	29 (47.5)
Surgical approach		
Laparoscopic/thoracoscopic	32 (54.2)	31 (50.8)
Laparoscopic/thoracoscopic assisted	7 (11.9)	8 (13.1)
Open	20 (33.9)	22 (36.1)
Anaesthetic maintenance		
Propofol	7 (11.9)	10 (16.4)
Volatile	46 (78.0)	47 (77.0)
Propofol + volatile	6 (10.2)	4 (6.6)
Antiemetic prophylaxis	51 (86.4)	53 (86.9)
Regional analgesia	17 (28.8%)	18 (29.5%)
Neuromuscular blocking drug		
Rocuronium	49 (83.1)	49 (80.3)
Vecuronium	10 (16.9)	13 (21.3)
Neuromuscular monitoring		
Quantitative ± qualitative	28 (47.5)	26 (42.6)
Qualitative alone	27 (45.8)	28 (45.9)
No monitoring	4 (6.8)	7 (11.5)
Extubated in operating room	57 (96.6)	61 (100.0)
TOF count at reversal	2.0 (2.0–4.0)	4.0 (2.0–4.0)
Sugammadex	56 (94.9)	2 (3.3)
Dose (mg)	200 (200–200)	200 (0.0–200.0)
Neostigmine	-	59 (96.7)
Dose (mg)	-	2.5 (2.5–2.5)
TOF ratio at extubation	1.04 (0.94–1.10)	0.93 (0.88–1.00)
Time from reversal to extubation (min)	9.0 (5.5–14.0)	10.0 (7.0–17.0)
Further reversal	-	9 (15.0)
Sugammadex	-	5 (55.6)
Neostigmine	-	4 (44.4)
Duration of anaesthesia (h)	3.8 (2.8–5.4)	4.0 (2.7–5.0)

Results are presented as median (interquartile range) and number (percent). *TOF* train of four. Missing data: TOF count = 19 (15.8%), TOF ratio = 32 (26.7%), time from reversal to extubation = 3 (2.5%) and further reversal = 3 (2.5%). Of 59 participants randomised to sugammadex, 2 were not extubated and 1 received no reversal. Of 61 participants randomised to neostigmine, 2 were administered sugammadex

Postoperative characteristics are presented in Table 3 and the Additional Information. The median duration of post anaesthesia care unit stay was 1.1 (0.8–1.7) h. No further reversal or airway management was required in the post anaesthesia care unit. Postoperative nausea and vomiting was experienced by 56 (47%) of patients from post anaesthesia care unit admission until postoperative day 1. Five (4.2%) of patients had an unplanned intensive care unit/high dependency unit admission. The median duration of hospital stay was 5.1 (3.2–8.4) days.

**Table 3:** Post anaesthesia care unit characteristics

Characteristic	Sugammadex (***n*** = 59)	Neostigmine (***n*** = 61)
*PACU*		
Direct ICU/HDU transfer	2 (3.4)	1 (1.6)
Airway management in PACU	0	0
Duration of PACU stay (h)	1.2 (0.8–2.0)	1.1 (0.7–1.6)
Discharge destination from PACU		
Ward	50 (87.7)	54 (90.0)
ICU/HDU	7 (12.3)	6 (10.0)
Acceptability of protocol		
Highly acceptable	32 (56.1)	25 (42.4)
Acceptable	24 (42.1)	27 (45.8)
Neutral	0 (0.0)	3 (5.1)
Unacceptable	1 (1.8)	3 (5.1)
Highly unacceptable	0 (0.0)	1 (1.7)
*Postoperative day 1*		
PONV since PACU arrival	26 (44.1)	30 (49.2)
Anti-emetic since PACU arrival	25 (42.4)	34 (55.7)
QoR-15	101.2 (18.0)	102.6 (14.5)
Change in QoR-15 (day 1 - preoperative)	−30.3 (21.0)	−34.3 (18.3)
EQ-5D-5L score	0.4 (0.1–0.6)	0.4 (0.1–0.5)
EQ-VAS score	60.0 (50.0–80.0)	70.0 (60.0–75.0)
*Hospital discharge*		
Unplanned ICU/HDU admission	3 (5.1)	2 (3.3)
Duration of hospital stay (days)	5.1 (3.1–9.2)	5.1 (3.2–8.2)
Primary outcome—adjudicated	5 (8.5)	5 (8.2)
Atelectasis	5 (8.5)	5 (8.2)
*Postoperative day 30*		
Days alive and at home	25.0 (19.0–27.0)	24.0 (21.0–27.0)
*Postoperative 3 months*		
EQ-5D-5L score	0.9 (0.8–1.0)	0.9 (0.8–1.0)
EQ-VAS score	80.0 (65.0–86.0)	80.0 (70.0–90.0)
Frailty		
Non-frail (CFS score = 1–4)	55 (94.8)	56 (96.6)
Frail (CFS score = 5–9)	3 (5.2)	2 (3.4)

Results are presented as mean (standard deviation), median (interquartile range), and number (percent). *CFS* Clinical Frailty Scale, *EQ-5D-5L* EuroQol 5-dimension 5-level, *EQ-VAS* EuroQol–Visual Analogue Scale, *HDU* high dependency unit, *ICU* intensive care unit, *PACU* post-anaesthesia care unit, *QoR* quality of recovery. Missing data: duration of PACU stay = 3 (2.5%), discharge destination from PACU = 3 (2.5%) (these patients admitted directly to ICU); acceptability of protocol = 4 (3.3%), CFS = 4 (3.3%); postoperative 3 months EQ-5D-5L/EQ-VAS = 4 (3.3%)

Median EQ-5D-5L scores were 0.9 (0.9–1.0) at baseline, 0.4 (0.1–0.5) on postoperative day 1 and 0.9 (0.8–1.0) at 3 months postoperatively. Median EQ-VAS scores were 80 (75–90) at baseline, 65 (50–77.5) on postoperative day 1 and 80 (70–90) at 3 months postoperatively (Tables 1 and 3). A sensitivity analysis revealed similar results when the English value set was used for the Australian data (Additional Information).

### Pilot outcomes

The primary outcomes was observed in 5 (8.5%) of the 59 patients in the sugammadex group and 5 (8.2%) of the 61 patients in the neostigmine group (OR 1.02, 95% CI 0.28 to 3.67, *p* = 0.976) (Table 4). There were no significant differences in any secondary outcomes between the two groups. Safety outcomes and adverse events are presented in the Additional Information.

**Table 4:** Pilot outcomes

Outcome	Estimate (95% confidence interval)	***p*** value
*Primary outcome—adjudicated*		
New pulmonary complications up to hospital discharge (or postoperative day 7 if still in hospital)	1.02 (0.28 to 3.67)	0.976
*Secondary outcomes*		
Duration of PACU stay (h)	0.20 (−0.07 to 0.47)	0.152
PONV on postoperative day 1	0.80 (0.38 to 1.71)	0.565
QoR-15 on postoperative day 1	−0.62 (−6.28 to 5.05)	0.830
Days alive and at home on postoperative day 30	0.00 (−2.15 to 2.15)	1.000
Frailty at 3 months postoperatively	1.68 (0.27 to 10.49)	0.579

*CFS* Clinical Frailty Score, *PACU* post-anaesthesia care unit, *PONV* postoperative nausea and vomiting. *QoR* quality of recovery, *Frailty* CFS >4. Estimates of the treatment effect are presented as odds ratios (primary outcome, PONV and frailty), absolute mean (QoR-15) or absolute median (duration of PACU stay and days alive and at home on postoperative day 30) differences. All analyses are adjusted for site. Frailty and QoR-15 are also adjusted for preoperative values. Missing data: duration of PACU stay = 3 (2.5%), CFS at 3 months postoperatively = 4 (3.3%)

## Discussion

We have demonstrated the feasibility of a large international randomised controlled trial of sugammadex, neostigmine and postoperative pulmonary complications in adult patients having abdominal and intrathoracic surgery. Our recruitment rate (80%) and rate of administration of the randomised intervention without crossover (98%) were high and our 3-month loss to follow-up rate (3.3%) was low. Case report forms were complete at 3 months in all the remaining patients. The protocol was acceptable or highly acceptable to the anaesthetist in 93% of cases. The median time taken for trial procedures was 3.5 h. No specific barriers to trial processes were encountered.

Opportunities to improve processes were identified. More guidance about neuromuscular monitoring and reversal among anaesthetists and trial coordinators is required, as quantitative neuromuscular monitoring was only applied in 45% of the patients, and train-of-four counts and train-of-four ratios were only recorded using either quantitative or qualitative monitoring in 84% and 73% of patients, respectively. However, in patients where neuromuscular monitoring was used, median train-of-four counts at reversal (3.0 [2.0–4.0]) and median train-of-four ratios at extubation (0.97 [0.91–1.10]) were consistent with guidelines. We also identified the need to carefully explain the rationale for randomisation at the end of surgery, the aim of which was to avoid biased anaesthetic management and to incentivise careful management of neuromuscular blockade. Areas for improvement of the paper case report form and database were identified, which will inform database development for the main trial. We will also eliminate fields now deemed unnecessary (e.g., anti-muscarinic drug doses).

We demonstrated that we can recruit patients who are at risk of postoperative pulmonary complications (70% of patients were considered at intermediate or high risk). The cohort was also at relatively high risk of postoperative nausea and vomiting (43.3% had an Apfel score of 3–4). The sample size was too small to draw firm conclusions about the effect of sugammadex versus neostigmine on the primary or secondary outcomes.

A strength of this feasibility and pilot trial is that it was conducted in two regions, each with different patient characteristics and healthcare systems. We included a rural site with experienced trial coordinators who were new to anaesthesia clinical trials. The pilot trial protocol, operating procedures and case report form were blueprinted on draft documents for the main trial. As we did not identify the need for any major changes, our trial processes therefore will be generalisable.

Limitations of our trial include that it was conducted during a period when surgical activity was affected by the coronavirus pandemic, by both reductions in elective surgery activity and elective surgery surges. We believe that similar or better recruitment can be achieved after the pandemic ends. We only included sites in one state of Australia and did not include any sites in New Zealand, although these regions have similar patient populations and healthcare systems to Victoria. Finally, the sample size of this trial was appropriate for testing our feasibility aims, but insufficient to support drawing conclusions about treatment effects.

## Conclusions

We demonstrated the feasibility of a large international randomised controlled trial of sugammadex, neostigmine and postoperative pulmonary complications in adult patients having abdominal and intrathoracic surgery. Collection of patient-reported quality of life measurements necessary to support cost-effectiveness evidence for Health Technology Appraisal is also feasible. The burden of postoperative pulmonary complications on patients, families and healthcare systems is very significant but few proven preventive measures are available. A large definitive trial of sugammadex, neostigmine and postoperative pulmonary complications is urgently required.

## Supplementary Information

**Additional file 1.**

## Data Availability

Available from the authors on request (see https://www.anzctr.org.au for details).

## Abbreviations

aOR: Adjusted odds ratio
ARISCAT: Assess Respiratory Risk in Surgical Patients in Catalonia
CFS: Clinical Frailty Scale
CI: Confidence interval
EQ-VAS: EuroQol-Visual Analogue Scale
QoR-15: Quality of Recovery-15
