# Supplementary material for: Sugammadex, neostigmine and postoperative pulmonary complications: an international randomised feasibility and pilot trial
Source: Pilot Feasibility Stud. 2021 Nov 9;7:200. doi: 10.1186/s40814-021-00942-9 (PMC8576081; doi:10.1186/s40814-021-00942-9)
Supplement: Supplementary file 1 — Additional file 1. [file 40814_2021_942_MOESM1_ESM.docx]

**Sugammadex, neostigmine and postoperative pulmonary complications: an international randomised feasibility and pilot study**

**Additional information**

Contents

[Postoperative pulmonary complications 2](#_Toc85515236)

[Trial coordinator interview results 4](#_Toc85515237)

[Additional table 1: Baseline characteristics 7](#_Toc85515238)

[Additional table 2: Intraoperative characteristics 8](#_Toc85515239)

[Additional table 3: Postoperative characteristics 9](#_Toc85515240)

[Additional table 4: Safety outcomes (as per actual treatment) 10](#_Toc85515241)

[Additional table 5: Adverse events (as per actual treatment) 11](#_Toc85515242)

[Additional table 6: EQ-5D-5L sensitivity analysis 12](#_Toc85515243)

[References 13](#_Toc85515244)

# Postoperative pulmonary complications

Standardised Endpoints for Perioperative Medicine - Core Outcome Measures in Perioperative and Anaesthetic Care (StEP-COMPAC) definition [1]

*Mechanism*

Composite of respiratory diagnoses that share common pathophysiological mechanisms including pulmonary collapse and airway contamination:

1. Atelectasis detected on computed tomography or chest radiograph
2. Pneumonia using United States Centers for Disease Control criteria [2]
3. Acute respiratory distress syndrome using Berlin consensus definition [3]
4. Pulmonary aspiration (clear clinical history and radiological evidence)

*Severity*

1. None: planned use of supplemental oxygen or mechanical respiratory support as part of routine care, but not in response to a complication or deteriorating physiology. Therapies which are purely preventive or prophylactic, for example high flow nasal oxygen or continuous positive airway pressure should be recorded as none.
2. Mild: therapeutic supplemental oxygen <0.6 fraction inspired O_2_
3. Moderate: therapeutic supplemental oxygen ≥0.6 fraction inspired O_2_, requirement for high flow nasal oxygen, or both
4. Severe: unplanned non-invasive mechanical ventilation, continuous positive airway pressure or invasive mechanical ventilation requiring tracheal intubation

*Exclusions*

Other diagnoses that do not share a common biological mechanism are best evaluated separately and only when clearly relevant to the treatment under investigation:

1. Pulmonary embolism
2. Pleural effusion
3. Cardiogenic pulmonary oedema
4. Pneumothorax
5. Bronchospasm

*Pneumonia [2]*

Two or more serial chest radiographs with at least one of the following (one radiograph is sufficient for patients with no underlying pulmonary or cardiac disease)

1. New or progressive and persistent infiltrates
2. Consolidation
3. Cavitation

AND at least one of the following:

1. Fever (>38°C) with no other recognised cause
2. Leucopaenia (white cell count <4x10^9^/L) or leucocytosis (white cell count >12x10^9^/L)
3. For adults >70 years old, altered mental status with no other recognised cause

AND at least two of the following:

1. New onset of purulent sputum or change in character of sputum, or increased respiratory secretions, or increased suctioning requirements
2. New onset or worsening cough, or dyspnoea, or tachypnoea
3. Rales or bronchial breath sounds
4. Worsening gas exchange (hypoxaemia, increased oxygen requirement, increased ventilator demand)

Acute respiratory distress syndrome [3]

All four of the following criteria:

1. Timing: within 1 week of a known clinical insult or new or worsening respiratory symptoms
2. Chest imaging: bilateral opacities not fully explained by effusions, lobar/lung collapse or nodules
3. Origin of oedema: respiratory failure not fully explained by cardiac failure or fluid overload (requires objective assessment, e.g. echocardiography, to exclude hydrostatic oedema)
4. Oxygenation: mild PaO_2_:FiO_2_ between 26.7 and 40.0 kPa (200-300 mm Hg) with positive end expiratory pressure or continuous positive airway pressure ≥5 cm H_2_O; moderate PaO_2_:FiO_2_ between 13.3 and 26.6 kPa (100-200 mm Hg) with positive end expiratory pressure ≥5 cm H_2_O; severe PaO_2_: FiO_2_ ≤13.3 kPa (100 mm Hg) with positive end expiratory pressure ≥5 cm H_2_O.

# Trial coordinator interview results

| **Question** | **Site 1** | **Site 2** | **Site 3** | **Site 4** | **Site 5** |
| --- | --- | --- | --- | --- | --- |
| 1. *Please describe the governance approval process at your site for this pilot study. What additional help could have been made available by the trial office?* | No more help needed. No issues. Timeframes no different to usual. | Quickest submission ever (4 hours). Documents provided were complete and orderly. Governance approval slow because of the contract (divisional director, finance signoff required) and because of research office restructuring. | Easy. Good documents and relationship with research office. Contract process quick. | Had to submit a full ethics application as well as multiple language consent forms. Also needed to obtain insurance and regulatory approvals. Trial office was helpful in helping us to initiate the study. | The process was facilitated as we could refer to previous applications in our region. We also bought insurance before study initiation. Trial office was helpful in helping us to initiate the study. |
| 1. *Please describe your screening process for this pilot study. Were there any barriers to correctly identifying eligible patients?* | Preadmission clinic or cold call as first contact. Face to face at preadmission clinic or on day of surgery. No barriers. | Identifying patients was simple because inclusion criteria were simple. Most approached on day of surgery. | Got help from pre-admission clinic staff. Weekly and then more frequent meetings with them to go over patient bookings. Sent patients home from clinic with consent form. Sometimes received calls from anaesthetists. | Elective surgery lists screened the day before their surgery. Each day’s recruitment list checked with principal investigator. Screening criteria were clear. Last minute changes to surgical lists meant we needed to double check to make sure we didn’t miss patients. | Mainly screened abdominal surgery patients. Each day’s recruitment list checked with principal investigator. Screening criteria were easy to follow. Last minute changes to surgical lists meant we needed to double check to make sure we didn’t miss patients. |
| 1. *Please describe your experience of the patient information and consent form. Was it easy to explain the study to the patients? Were there any parts of the consent form that attracted questions or concern? What feedback did you receive from patients about provision of consent or withholding of consent?* | Easy to explain. Sometimes mention of medications frightening to patients, but no more so than usual with other studies. Patients liked it because all completed in theatre, no extra visits or blood tests. No parts of the consent form caused concern. Some of the patients (e.g., cancer, quick path from diagnosis to surgery) had a general problem with research but not a specific one with this study. | Easy to explain. Both interventions in wide use. Just need evidence. One patient asked “Am I going to wake up?”. No other specific issues raised. Patients who refused were saying no to research in general and not this study. Some found it all too hard. One or two older patients with lung issues said no. | Consent new to trial coordinators as they come from a different speciality background. Easy. Nothing to sell as both groups were standard of care. Most patients keen to be involved. Anxious patients wouldn’t join any study (not this study in particular). Nothing in the consent form caused concern. | Consent forms were quite clear. Patients were generally satisfied with the information provided on it. We did not encounter much difficulty in obtaining consent. Some asked whether they would need to pay for sugammadex. Some patients wished to be assigned to sugammadex. Some patients refused as they preferred usual practice (i.e., neostigmine). | The consent form was laid out in a clear way that helped to explain the study to potential patients. Easy to explain. A few older patients did not understand reversal of neuromuscular blockade. Enlisted help of patients’ children. The most common study refusal reason was that they preferred their anaesthetist to do “whatever they usually do”, instead of being randomized. |
| 1. *Please describe your experience with the attending anaesthetists. Was it easy to explain the study to the anaesthetists? What feedback did you receive from the anaesthetists about the trial procedures?* | Easy. A lot were open minded about the study. Need a better ‘cheat sheet’ for anaesthetists. The thoracic anaesthetists were mainly happy. Some reluctance for thoracic robotic surgery. Some reluctance due to time pressures, obesity, renal function. A few anaesthetists were tired, didn’t want interruptions to workflow. Anaesthetists were happier when patient randomised to sugammadex. | One very clear that they did not like randomisation at the end. Others ranged from receptive to not interested but on the whole pretty helpful. Some asked hasn’t this already been answered? Similar response to past trials. Some people do not routinely use reversal so were unhappy but still helped. | No anaesthetist declined. Some more enthusiastic than others. Registrars delighted. Some slow on completing the acceptability question. Anaesthetists happier when randomised to sugammadex. | In general, easy to explain. Instructions sheets prepared to be passed to the anaesthetist near the end of the operation. Regarding rocuronium dosage, the most common question was whether an infusion is a must. | Initiating meeting and instructions on the day for anaesthetists with reminders by operating theatre nurses. Some anaesthetists wished to know the assigned group at the beginning of the surgery instead of right before the reversal. |
| 1. *Please describe your experience with the case report form. Were any fields unclear or very time-consuming to complete? Did you need help from the trial office to fill in the case report form?* | Case report form and database not as user friendly as other trials. Not enough logic to ease entry and prevent mistakes. No specific issues with any data points. | Randomisation form was on reversal page. Just different and not a problem. Paper case report form and database were not the same. Anaesthetists made a mess of the case report form. Compulsory entry of inclusion and exclusion criteria before randomisation made randomising in a rush difficult. Discharged from hospital: why is it on the 30-day and 3-month form as well as the discharged form? Safety endpoint form: can only capture each type once. Not too much work looking for safety outcomes. | Questions about 30-day follow-up and infections were answered. Better education about neuromuscular monitoring required. Worried about whether survey results immediately preoperatively are valid because of such a short period to develop rapport with the patients. Would self administration be more accurate? Then you have problems with completion. | The case report form was quite self-explanatory. Formatting of the case report form could have been clearer but was good enough for pilot study. It would also be great if the paper case report form and web case report form had the same formatting. | Same issues with case report form and database as the other site in this region. |
| 1. *Please describe your experience with contacting patients after they went home. Were there any issues with collecting data over the phone?* | No problems with follow-ups. No problems with administering surveys on the phone. | One grumpy patient – unusual because patients are usually nice. A couple could not be contacted – just unlucky and nothing to do with this study. Consent form included contacting general practitioners which was good. Need more training with the Clinical Frailty Scale. Hard to discriminate between disease activity and physical activity. | A bit of an issue because calling from a private number. But once contacted friendly after that. | Patients usually answered our phone call within 1-3 calls. Some patients forgot they were in a study trial. Other than that, our patients in general were cooperative with our follow-up phone calls. | The patients at our hospital are in general older. Some difficulty with phone interviews, requiring help from family members. |

# Additional table 1: Baseline characteristics

| **Characteristic** | **Sugammadex (n = 59)** | **Neostigmine (n = 61)** |
| --- | --- | --- |
| Weight (kg)) | 70.8 (17.0) | 75.8 (18.0) |
| Height (cm) | 165.6 (9.6) | 167.4 (9.3) |
| Ethnicity | 1 (0.8) | 0 (0.0) |
| Aboriginal | 0 (0.0) | 1 (1.6) |
| European | 26 (44.1) | 29 (47.5) |
| Asian | 32 (54.2) | 30 (49.2) |
| South Asian | 0 (0.0) | 1 (1.6) |
| Peoples of the Americas | 1 (1.7) | 0 (0.0) |
| Residence (home) | 59 (100.0) | 61 (100.0) |
| Smoker |  |  |
| Current daily smoker | 3 (5.1) | 5 (8.2) |
| Current most less than daily | 1 (1.7) | 3 (4.9) |
| Ex-daily smoker or ≥100 cigarettes | 27 (45.8) | 24 (39.3) |
| Never smoker (<100 cigarettes) | 28 (47.5) | 29 (47.5) |
| Asthma | 3 (5.1) | 7 (11.5) |
| COPD | 4 (6.8) | 3 (4.9) |
| Obstructive sleep apnoea |  |  |
| No | 55 (93.2) | 56 (91.8) |
| Confirmed | 3 (5.1) | 3 (4.9) |
| Suspected | 1 (1.7) | 2 (3.3) |
| Respiratory infection | 0 (0.0) | 0 (0.0) |
| Heart failure | 1 (1.7) | 2 (3.3) |
| Diabetes mellitus | 13 (22.0) | 10 (16.4) |
| PONV | 8 (13.6) | 2 (3.3) |
| Motion sickness | 6 (10.2) | 3 (4.9) |
| Oxygen saturation (%) | 97.0 (96.0-98.0) | 97.0 (96.0-98.0) |
| Haemoglobin (g/dL) | 135.0 (120.0-140.0) | 134.0 (120.0-150.0) |
| eGFR (mL/min/1.73m^2^) | 83.0 (65.0-90.0) | 88.0 (75.0-91.0) |
| Albumin (g/L) | 38.0 (34.5-40.5) | 38.0 (34.0-40.0) |
| HbA1C (%) | 5.9 (5.3-6.7) | 6.2 (5.4-7.1) |
| CFS score | 2.0 (2.0-3.0) | 2.0 (2.0-3.0) |

Results are presented as mean (standard deviation), median (interquartile range) or number (percent). CFS = Clinical Frailty Scale. COPD = chronic obstructive pulmonary disease. eGFR = estimated glomerular filtration rate. HbA1c = haemoglobin A1C. PONV = postoperative nausea and vomiting; QoR = quality of recovery. Missing data: eGFR = 1 (0.8%); Albumin = 21 (17.5%); HbA1C = 73 (60.8%).

# Additional table 2: Intraoperative characteristics

| **Characteristic** | **Sugammadex (n = 59)** | **Neostigmine (n = 61)** |
| --- | --- | --- |
| Surgical type |  |  |
| Thoracic lung | 12 (20.3) | 9 (14.8) |
| Thoracic other | 1 (1.7) | 1 (1.6) |
| Upper gastrointestinal | 6 (10.2) | 5 (8.2) |
| Hepato-biliary | 12 (20.3) | 16 (26.2) |
| Lower gastrointestinal | 18 (30.5) | 16 (26.2) |
| Renal, adrenal, ureter, bladder, prostate | 5 (8.5) | 11 (18.0) |
| Gynaecological | 5 (8.5) | 2 (3.3) |
| Vascular | 0 (0.0) | 0 (0.0) |
| Other | 0 (0.0) | 1 (1.6) |
| Benzodiazepine | 11 (18.6) | 13 (21.3) |
| Opioid | 59 (100.0) | 61 (100.0) |
| Propofol induction | 58 (98.3) | 61 (100.0) |
| Type of volatile |  |  |
| Sevoflurane | 42 (80.8) | 43 (84.3) |
| Isoflurane | 1 (1.9) | 0 (0.0) |
| Other sedatives/analgesics |  |  |
| Nitrous oxide | 4 (6.8) | 1 (1.6) |
| Alpha-2 agonist | 6 (10.2) | 9 (14.8) |
| Ketamine | 19 (32.2) | 20 (32.8) |
| Lignocaine | 21 (35.6) | 16 (26.2) |
| Magnesium | 6 (10.2) | 1 (1.6) |
| Antiemetic prophylaxis |  |  |
| Dexamethasone | 27 (45.8) | 30 (49.2) |
| 5HT-3 antagonist | 39 (66.1) | 39 (63.9) |
| Droperidol | 3 (5.1) | 3 (4.9) |
| Regional |  |  |
| Neuraxial | 10 (58.8) | 10 (55.6) |
| Other | 7 (41.2) | 8 (44.4) |
| Fraction inspired oxygen | 0.4 (0.4-0.6) | 0.5 (0.4-0.6) |
| Positive end expiratory pressure (mm Hg) | 5.0 (5.0-5.0) | 5.0 (5.0-5.0) |
| Tidal volume (mL) | 435.0 (405.0-480.0) | 470.0 (410.0-515.5) |
| Respiratory rate (per min) | 13.0 (12.0-14.0) | 13.0 (12.0-14.0) |
| Rocuronium (mg) | 100.0 (65.0-120.0) | 100.0 (75.0-125.0) |
| Vecuronium (mg) | 14.0 (12.0-20.0) | 15.0 (10.0-18.0) |
| Depth of anaesthesia monitoring | 25 (42.4) | 26 (42.6) |
| Glycopyrrolate | 0 (0.0) | 29 (47.5) |
| Dose (mg) | 0.0 (0.0-0.0) | 400.0 (400.0-500.0) |
| Further sugammadex dose (mg) | - | 200.0 (200.0-200.0) |
| Further neostigmine dose (mg) | 0.0 (0.0-0.0) | 1.9 (1.3-2.5) |

Results are presented as median (interquartile range) or number (percent). Missing data: Type of volatile = 17 (14.2); Positive end expiratory pressure = 11 (9.2); tidal volume = 11 (9.2); Respiratory rate = 11 (9.2)

# Additional table 3: Postoperative characteristics

| **Characteristic** | **Sugammadex (n = 59)** | **Neostigmine (n = 61)** |
| --- | --- | --- |
| *PACU* |  |  |
| Antiemetic | 6 (10.2) | 9 (14.8) |
| Dexamethasone | 1 (16.7) | 0 (0.0) |
| 5HT-3 antagonist | 2 (33.3) | 8 (88.9) |
| Droperidol | 1 (16.7) | 0 (0.0) |
| Metoclopramide | 2 (33.3) | 1 (11.1) |
| *Postoperative day 1* |  |  |
| Anti-emetics | 25 (42.4) | 30 (49.2) |
| 5HT-3 antagonist | 21 (35.6) | 25 (41.0) |
| Droperidol | 1 (1.7) | 1 (1.6) |
| Metoclopramide | 4 (6.8) | 8 (13.1) |
| Cyclizine | 1 (1.7) | 1 (1.6) |
| *Discharge* |  |  |
| Discharge destination |  |  |
| Home | 57 (96.6) | 56 (91.8) |
| Residential aged care | 1 (1.7) | 0 (0.0) |
| Another hospital | 0 (0.0) | 2 (3.3) |
| Another location | 1 (1.7) | 3 (4.9) |
| *Postoperative 3 months* |  |  |
| CFS score | 3.0 (2.0-3.0) | 3.0 (2.0-3.0) |
| Change in CFS score (Day 1 - preoperative) | 0.0 (0.0-1.0) | 0.0 (0.0-1.0) |

CFS = Clinical Frailty Scale. PACU = post anaesthesia care unit. Results are presented as number (percent). Missing data: CFS = 4 (3.3%)

# Additional table 4: Safety outcomes (as per actual treatment)

| **Random Group** | **n** | **PACU** | **POD1** | **POD2** | **POD3** | **POD7** | **Discharge** | **TOTAL** |
| --- | --- | --- | --- | --- | --- | --- | --- | --- |
| Sugammadex | 58 | 1 | 13 | 10 | 6 | 6 | 8 | 44 (75.9) |
| Neostigmine | 59 | 1 | 4 | 3 | 3 | 6 | 10 | 27 (45.8) |
| **TOTAL** | **117** | **2** | **17** | **13** | **9** | **12** | **18** | **71** |

PACU = Post anaesthesia care unit; POD = postoperative day. Results are presented as number of events or total number of events (percent by patients who received that treatment). Excludes participants who did not receive either of the randomised interventions. Infectious, cardiovascular, thromboembolic, respiratory, neurological, digestive, renal, musculoskeletal and allergic untoward events occurring from randomisation until hospital discharge (or postoperative day 7 if still in hospital).

# Additional table 5: Adverse events (as per actual treatment)

| **Random Group** | **n** | **OR** | **PACU** | **POD1** | **POD2** | **POD3** | **POD7** | **Discharge** | **30 days** | **3 months** | **TOTAL** |
| --- | --- | --- | --- | --- | --- | --- | --- | --- | --- | --- | --- |
| Sugammadex | 58 | 0 | 0 | 1 | 0 | 1 | 1 | 0 | 8 | 2 | 13 (22.4) |
| Neostigmine | 59 | 0 | 0 | 1 | 1 | 1 | 0 | 0 | 11 | 4 | 18 (30.5) |
| **TOTAL** | **117** | **0** | **0** | **2** | **1** | **2** | **1** | **0** | **19** | **6** | **31** |

OR = operating room; PACU = post anaesthesia care unit; POD = postoperative day. Results are presented as number of events or total number of events (percent by patients who received that treatment). Excludes participants who did not receive either of the randomised interventions. Adverse events were collected from randomisation until 3 months postoperatively.

# Additional table 6: EQ-5D-5L sensitivity analysis

| **Characteristic** | **Total (n = 120)** | **Sugammadex (n = 59)** | **Neostigmine (n = 61)** |
| --- | --- | --- | --- |
| *Baseline* |  |  |  |
| EQ-5D-5L | 0.9 (0.9-1.0) | 0.9 (0.8-1.0) | 0.9 (0.9-1.0) |
| EQ-5D-5L (sensitivity analysis) | 0.9 (0.9-1.0) | 0.9 (0.8-1.0) | 0.9 (0.9-1.0) |
| *Postoperative day 1* |  |  |  |
| EQ-5D-5L | 0.4 (0.1-0.5) | 0.4 (0.1-0.6) | 0.4 (0.1-0.5) |
| EQ-5D-5L (sensitivity analysis) | 0.5 (0.2-0.6) | 0.5 (0.2-0.6) | 0.5 (0.2-0.6) |
| *Postoperative 3 months* |  |  |  |
| EQ-5D-5L | 0.9 (0.8-1.0) | 0.9 (0.8-1.0) | 0.9 (0.8-1.0) |
| EQ-5D-5L (sensitivity analysis) | 0.9 (0.8-1.0) | 0.9 (0.8-1.0) | 0.9 (0.8-1.0) |

Results are presented as median (interquartile range). Missing data: postoperative 3 months = 4 (3.3%). EQ-5D-5L index values for Australia were computed using the United States value set [4]. As a sensitivity analysis the EQ-5D-5L index values for Australia were computed using the English value set [5]. EQ-5D-5L index values for Hong Kong were computed using the Hong Kong value set [6].

# References

1. Abbott TEF, Fowler AJ, Pelosi P, Gama de Abreu M, Moller AM, Canet J, et al. A systematic review and consensus definitions for standardised end-points in perioperative medicine: pulmonary complications. Br J Anaesth. 2018;120(5):1066-79.

2. Horan TC, Andrus M, Dudeck MA. CDC/NHSN surveillance definition of health care-associated infection and criteria for specific types of infections in the acute care setting. Am J Infect Control 2008;36(5):309-32.

3. Ranieri VM, Rubenfeld GD, Thompson BT, Ferguson ND, Caldwell E, Fan E, et al. Acute respiratory distress syndrome: the Berlin Definition. Jama. 2012;307(23):2526-33.

4. Pickard AS, Law EH, Jiang R, Pullenayegum E, Shaw JW, Xie F, et al. United States valuation of EQ-5D-5L health states using an international protocol. Value Health. 2019;22(8):931-41.

5. Devlin NJ, Shah KK, Feng Y, Mulhern B, van Hout B. Valuing health-related quality of life: An EQ-5D-5L value set for England. Health Econ. 2018;27(1):7-22.

6. Wong ELY, Ramos-Goñi JM, Cheung AWL, Wong AYK, Rivero-Arias O. Assessing the use of a feedback module to model EQ-5D-5L health states values in Hong Kong. Patient. 2018;11(2):235-47.
